# Designing Highly Precise Overlay Targets for Asymmetric Sidewall Structures Using Quasi-Periodic Line Widths and Finite-Difference Time-Domain Simulation

**DOI:** 10.3390/s23094482

**Published:** 2023-05-04

**Authors:** Hung-Chih Hsieh, Meng-Rong Wu, Xiang-Ting Huang

**Affiliations:** Department of Electro-Optical Engineering, National United University, No. 2 Lienda, Miaoli 36063, Taiwan

**Keywords:** overlay measurement, asymmetric profile, finite-difference time-domain simulation, diffraction-based overlay, overlay target design

## Abstract

The present study introduces an optimized overlay target design to minimize the overlay error caused by asymmetric sidewall structures in semiconductor manufacturing. To achieve this goal, the overlay error formula was derived by separating the asymmetric bottom grating structure into symmetric and asymmetric parts. Based on this formula, it was found that the overlay target design with the linewidth of the bottom grating closed to the grating period could effectively reduce the overlay error caused by the sidewall asymmetry structure. Simulation results demonstrate that the proposed design can effectively control the measurement error of different wavelengths within ±0.3 nm, even under varying sidewall angles and film thicknesses. Overall, the proposed overlay target design can significantly improve the overlay accuracy in semiconductor manufacturing processes.

## 1. Introduction

Overlay (OVL) measurement has become a significant challenge in lithography for advanced technology nodes in semiconductor manufacturing [1,2,3]. As per the report of the International Roadmap for Devices and System 2020, the overlay tolerance of a scanner decreases as the semiconductor process node advances. The overlay tolerance is typically 1/5 to 1/10 of the minimum feature size. For instance, the transistor gate pitch for the 5 nm process node is around 50 nm [4], and the gate width is around 20 nm. Consequently, the allowable OVL value is approximately 3.6 nm in the case of dynamic random-access memory (DRAM) components, and the associated OVL measurement accuracy is 0.36 nm, i.e., one tenth of the OVL value [3].

Overlay metrology can be broadly classified into two approaches: image-based overlay measurement (IBO) [5] and diffraction-based overlay measurement (DBO) [6,7,8,9,10,11,12,13,14]. IBO is a technique that relies on the use of optical microscopes to measure the alignment accuracy of successive layers of a semiconductor wafer. This technique involves capturing images of patterns on the layers and comparing them to the patterns on the previous layer. The alignment accuracy can be determined by analyzing the differences between the images. Moreover, the IBO technique requires that the alignment marks are visible and distinguishable between the layers. The alignment marks can be created by using a specific pattern in the design of the semiconductor chip, which allows for precise measurements to be taken. On the other hand, according to the DBO measurement principle, the OVL measurement signal is an asymmetric diffraction signal generated by the measurement target, and a linear calculation model calculates the OVL value. A more detailed explanation of the DBO principle will be provided in the Principle section.

Currently, DBO is widely used for overlay measurement in advanced technology nodes. According to the DBO measurement principle, the overlay measurement signal is an asymmetric diffraction signal generated by the measurement target, and a linear calculation model calculates the overlay value. However, during the manufacturing process, when the measurement target is processed with the etch process and the chemical–mechanical polish (CMP) process [5,15], the target topography changes into an asymmetric structure. At this stage, the intensity of the positive/negative first-order diffracted light produced by the asymmetric measurement target differs from the intensity of the diffracted light produced by a perfectly symmetric measurement target. This difference in the two sets of first-order diffracted light intensity results in errors in measuring the overlay value [16,17].

To reduce the measurement overlay error, many researchers have proposed several methods to improve the accuracy of the overlay measurement. Bhattacharyya et al. [18] proposed a multi-wavelength approach to improve the OVL measurement accuracy. The multi-wavelength measurement results will be significantly impacted if the number of available wavelengths is too few or the wavelength range is not wide enough. In addition, when measuring multiple wavelengths, it is necessary to measure data of different wavelengths simultaneously, so there will be a time delay in wavelength conversion, resulting in a significant increase in measurement time. Although the measured OVL error improved by 40% with the multi-wavelength approach, the time-consuming measurement to collect all the required multi-wavelength data was not acceptable in the in-line production of a semiconductor manufacturing factory. Shi et al. [19] used a multi-objective optimization genetic algorithm to optimize the overlay target with sidewall angle asymmetry of the bottom grating. This methodology provided a good guideline for designing the overlay targets for every different film stack. However, the overlay target design was specific to a particular film stack, which may not be suitable for other film stacks. The overlay target design is based on the properties of the specific film stack used during the design process, such as the thicknesses and refractive indices of the films. If the film stack is changed, the properties of the films will be different, and the previously designed overlay target may no longer be effective. Consequently, the overlay needs to be redesigned for the new film stacks and is time-consuming in the manufacturing process. In our previous study [20], we proposed a robust and wafer-less wavelength selection methodology by minimum asymmetry factor variation with finite-difference time-domain (FDTD) simulation. The maximum overlay error of the optimized wavelength selected by this method for a tested structure was −0.11 nm. However, the measurement wavelength needed to be re-optimized when the film stack changed due to semiconductor process variation, and for mass production, this is time-consuming.

The content of this paper builds upon previous research [20], where a robust and wafer-less wavelength selection methodology was proposed. The derived overlay calculation formula for the DBO measurement target with a symmetric bottom grating structure is also based on the previous work. In this paper, we take a step further by proposing a DBO overlay target design that can effectively minimize the overlay error caused by the asymmetric sidewall structure and that is robust for large film thickness variations. To achieve this goal, we separate the asymmetric structure into two parts and deduce the overlay error term caused by the asymmetric part. The only workable solution for minimizing the overlay error is identified, which is to make the linewidth of the bottom grating of the overlay target to be close to the grating period. The proposed method is verified by FDTD simulation, and the simulation results show that the measurement error of different wavelengths can be controlled within ±0.3 nm under different sidewall angles and film thickness variations.

## 2. Principle

### 2.1. Principle of Diffraction-Based Overlay (DBO) Measurement

**Figure 1:**
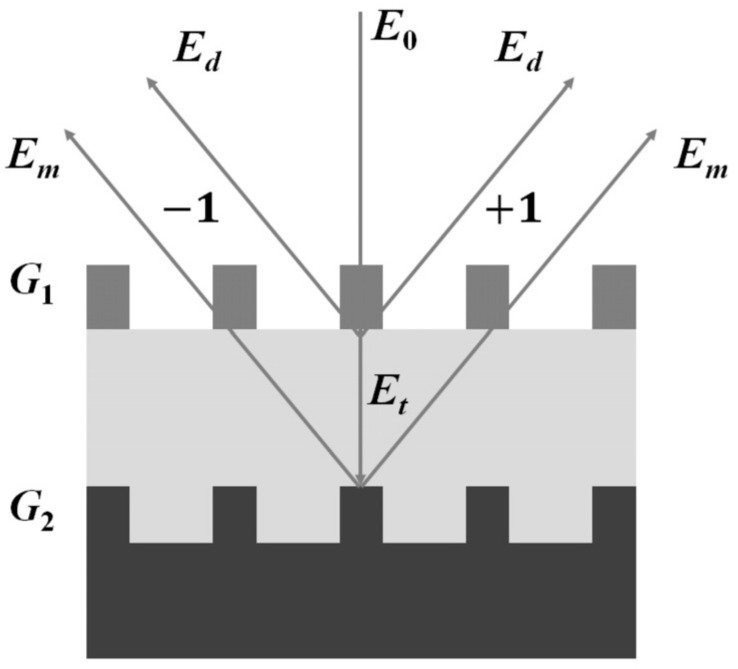
The basic optical configuration and target design for the DBO measurement.

The basic optical configuration and target design for the DBO measurement is shown in Figure 1. For convenience, the z-axis is chosen along the light propagation direction, and the y-axis is along the vertical direction. The DBO measurement target consists of the top grating structure ($G_{1}$), the middle-film structure, and the bottom grating structure ($G_{2}$). For simplicity, only one middle-film structure was used for deriving the DBO measurement principle.

The incident light $E_{0}$ is linearly polarized with a wavelength of λ, and the polarization direction of 45° with respect to the *x*-axis was divided into transmitted light $E_{t}$ and diffracted light $E_{d}$ by the top grating structure. According to the Jones matrix calculation [21,22], we can calculate the electric fields of $E_{t}$ and $E_{d}$, and they can be written as

(1) $$E_{t}=T_{G1}\cdot E_{0}=\left(\begin{array}{c} t_{G1}e^{i\varphi_{G1}}\\ t_{G1}e^{i\varphi_{G1}} \end{array}\right),$$

and

(2) $$E_{d}=R_{G1}\cdot E_{0}=\left(\begin{array}{c} r_{G1}e^{i\varphi_{G1}}\\ r_{G1}e^{i\varphi_{G1}} \end{array}\right),$$

where $t_{G1}$ and $r_{G1}$ are the transmission and reflection coefficients of the top grating, respectively; $\varphi_{G1}$ is the phase shift induced by the top grating, and it can be written as [23,24,25,26]

(3) $$\varphi_{G1}=-m\cdot \frac{2\pi S_{G1}}{P_{G1}},$$

where m is the diffraction order, $S_{G1}$ is the position of the top grating, and $P_{G1}$ is the period of the top grating. Substituting m=0 for the transmitted electric field $E_{t}$, we can obtain

(4) $$E_{t}=\left(\begin{array}{c} t_{G1}\\ t_{G1} \end{array}\right).$$

Then, the transmitted light $E_{t}$ passes through the middle-film structure and is diffracted by the bottom grating structure. The diffracted light then passes through the middle-film structure and the top grating again. Hence, we can calculate the electric field as follows:

(5) $$E_{m}=T_{G1}\cdot T_{f}\cdot R_{G2}\cdot T_{f}\cdot E_{t}=\left(\begin{array}{c} t_{G1}^{2}r_{G2}t_{f}^{2}e^{i(\varphi_{G2}+2\varphi_{f})}\\ t_{G1}^{2}r_{G2}t_{f}^{2}e^{i(\varphi_{G2}+2\varphi_{f})} \end{array}\right),$$

where $r_{G2}$ is the reflection coefficient of the bottom grating structure; $t_{f}$ and $\varphi_{f}$ are the transmission coefficient and the phase shift of the middle-film structure, respectively; and $\varphi_{G2}$ is the phase shift induced by the bottom grating, and it can be written as

(6) $$\varphi_{G2}=-m\cdot \frac{2\pi S_{G2}}{P_{G2}},$$

where $S_{G2}$ is the position of the bottom grating; $P_{G2}$ is the period of the bottom grating. Since the grating period of the top and bottom grating structures is the same in the general DBO target design, the positive first-order diffracted light diffracted by the top and bottom grating structures interferes, and so does the negative first-order diffracted light. Hence, we can calculate the electric field of the interference light, and it can be written as

(7) $$E_{inter}=E_{d}+E_{m}=\left(\begin{array}{c} r_{G1}e^{i\varphi_{G1}}+t_{G1}^{2}r_{G2}t_{f}^{2}e^{i(\varphi_{G2}+2\varphi_{f})}\\ r_{G1}e^{i\varphi_{G1}}+t_{G1}^{2}r_{G2}t_{f}^{2}e^{i(\varphi_{G2}+2\varphi_{f})} \end{array}\right).$$

Consequently, the intensity of the interference signal can be derived as

(8) $$I={\left|E_{d}+E_{m}\right|}^{2}=I_{DC}+\gamma \times \cos\left(\beta +\varphi_{G1}-\varphi_{G2}\right),$$

where $I_{DC}$ and γ are the DC term and amplitude of the interference signal, respectively, and they can be written as

(9) $$I_{DC}=2\left(r_{G1}^{2}+t_{G1}^{4}r_{G2}^{2}t_{f}^{4}\right),$$

and

(10) $$\gamma =4t_{G1}^{2}r_{G1}r_{G2}t_{f}^{2},$$

and $\beta =-2\varphi_{f}$ is the phase term, which is a function of the material properties (i.e., the refractive index and the film thickness) of the middle-film structure. Substituting Equations (3) and (6) into Equation (8) and replacing the grating periods by $P_{G1}=P_{G2}=P$, we can re-write the interference signal as

(11) $$I=I_{DC}+\gamma \times \cos\left(\beta +m\frac{2\pi }{P}OVL\right),$$

where $OVL=S_{G2}-S_{G1}$ is defined as the relative position of the top and bottom gratings.

The interference intensity of the positive and negative first-order diffracted light can be expressed as

(12) $$I^{+1}=I_{DC}+\gamma \times cos\left(\beta +\frac{2\pi }{P}OVL\right),$$

and

(13) $$I^{-1}=I_{DC}+\gamma \times cos\left(\beta -\frac{2\pi }{P}OVL\right).$$

We define the asymmetric interference intensities AI of the two first-order diffraction lights as follows:

(14) $$AI=I^{+1}-I^{-1}=-2\gamma \times sin\left(\beta \right)\times sin\left(\frac{2\pi }{P}OVL\right).$$

Since the *OVL* value is a few nanometers, and the period of the grating is several hundred nanometers, we can approximate the $sin\left(2\pi \cdot OVL/P\right)\approx 2\pi \cdot OVL/P$. Then, we can simplify Equation (14) to be

(15) AI=K×OVL,

where $K=-2\gamma \times sin\left(\beta \right)\times 2\pi /P$. Furthermore, since the OVL value is not equal to zero, it will cause the intensity difference between $I^{+1}$ and $I^{-1}$. To obtain the OVL value, two fixed and opposite displacements, +d and −d, in the design between the top and bottom gratings are added. In this way, the measured AI can be expressed as follows:

(16) $$AI_{+d}=K\times \left(OVL+d\right),$$

and

(17) $$AI_{-d}=K\times \left(OVL-d\right).$$

From Equations (16) and (17), the OVL value can be obtained and expressed as

(18) $$OVL=\frac{AI_{+d}+AI_{-d}}{AI_{+d}-AI_{-d}}\times d.$$

### 2.2. Asymmetric Intensity Calculation for Sidewall Asymmetric Bottom Grating Structure by the Grating-Separation Model

**Figure 2:**
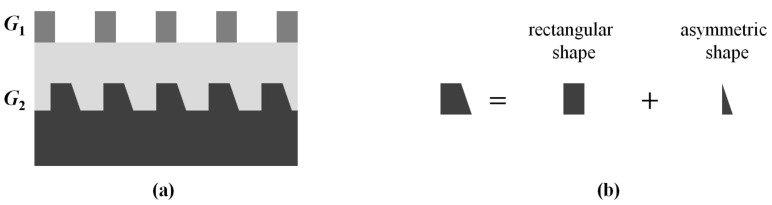
(**a**) The overlay target with asymmetric bottom grating structure; (**b**) separation of the asymmetric bottom grating structure.

In the perfect bottom grating structure that we discussed in Section 2.1, the profile of the bottom grating is a rectangular shape; that is, the grating profile is symmetrical. This section will discuss the sidewall asymmetric bottom grating profile structure, as shown in Figure 2a. The asymmetric bottom grating structure can be separated into two parts, such that one is the rectangular shape, and the other one is the asymmetric shape, as shown in Figure 2b.

Hence, the Jones matrix of $R_{G2}$ in Equation (5) can be modified by [27]

(19) $$R_{G2}^{\prime }=\left(\begin{array}{cc} r_{rect}e^{i\varphi_{rect}}+r_{asy}e^{i\varphi_{asy}} & 0\\ 0 & r_{rect}e^{i\varphi_{rect}}+r_{asy}e^{i\varphi_{asy}} \end{array}\right),$$

where $r_{rect}$, $\varphi_{rect}$, $r_{asy}$, and $\varphi_{asy}$ are the reflective coefficients and phase shifts of the rectangular part and asymmetric part of the bottom grating structure, respectively. Substituting Equation (19) into Equation (5), the modified electric field that is refracted by the bottom grating can be derived as

(20) $$E_{m}^{\prime }=T_{G1}\cdot T_{f}\cdot R_{G2}^{\prime }\cdot T_{f}\cdot E_{t}=\left(\begin{array}{c} t_{G1}^{2}r_{rect}t_{f}^{2}e^{i(\varphi_{rect}+2\varphi_{f})}+t_{G1}^{2}r_{asy}t_{f}^{2}e^{i(\varphi_{asy}+2\varphi_{f})}\\ t_{G1}^{2}r_{rect}t_{f}^{2}e^{i(\varphi_{rect}+2\varphi_{f})}+t_{G1}^{2}r_{asy}t_{f}^{2}e^{i(\varphi_{asy}+2\varphi_{f})} \end{array}\right).$$

The intensity of the interference signal can be derived as

(21) $$I={\left|E_{d}+E_{m}^{\prime }\right|}^{2}$$

$$=I_{DC}^{\prime }+\gamma_{1}\times \cos\left(\beta +\varphi_{G1}-\varphi_{rect}\right)$$

$$+\gamma_{2}\times \cos\left(\beta +\varphi_{G1}-\varphi_{asy}\right)$$

$$+\gamma_{3}\times \cos\left(\varphi_{asy}-\varphi_{rect}\right),\;$$

where $I_{DC}^{\prime }$ is the DC term, and $\gamma_{i}$ (i=1, 2, 3) is the amplitude of the interference signal. These items can be expressed as

(22) $$I_{DC}^{\prime }=2\left(r_{G1}^{2}+t_{G1}^{4}r_{rect}^{2}t_{f}^{4}+t_{G1}^{4}r_{asy}^{2}t_{f}^{4}\right),$$

(23) $$\gamma_{1}=4t_{G1}^{2}r_{G1}r_{rect}t_{f}^{2},$$

(24) $$\gamma_{2}=4t_{G1}^{2}r_{G1}r_{asy}t_{f}^{2},$$

and

(25) $$\gamma_{3}=4t_{G1}^{4}r_{asy}r_{rect}t_{f}^{4},$$

assuming that there is no asymmetric profile in the bottom grating. Hence, the $r_{asy}=0$, and thus $I_{DC}^{\prime }=I_{DC}$, $\gamma_{1}=\gamma$, $\gamma_{2}=0$, and $\gamma_{3}=0$. The intensity of the interference signal of Equation (21) is equal to Equation (8). This result could be evidence of the correctness of the grating-separation model methodology.

**Figure 3:**
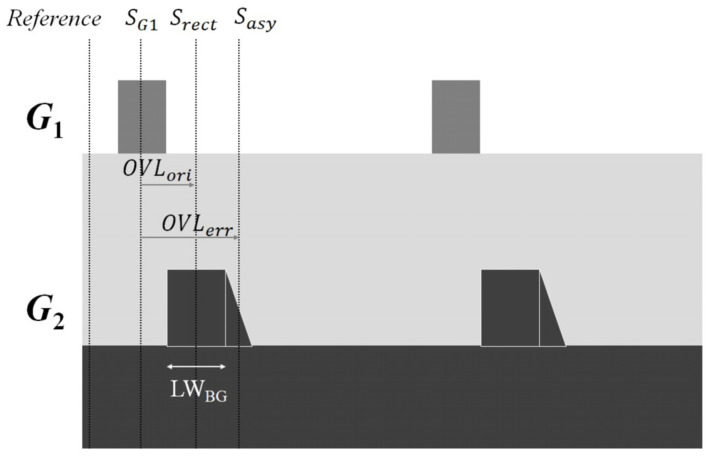
The basic optical configuration and target design for the DBO measurement.

Consequently, we can calculate the asymmetric interference intensity of the positive and negative first-order diffraction lights as

(26) $$AI^{\prime }=-2\gamma_{1}\times sin\left(\beta \right)\times sin\left(\frac{2\pi }{P}OVL_{ori}\right)-2\gamma_{2}\times sin\left(\beta \right)\times sin\left(\frac{2\pi }{P}OVL_{err}\right),$$

where $OVL_{ori}=S_{rect}-S_{G1}$ is the overlay between the top grating and the rectangular part of the bottom grating; $S_{rect}$ and $S_{G1}$ are the positions of the rectangular part of the bottom grating and the top grating to the reference point, respectively; the $OVL_{err}=S_{asy}-S_{G1}$ is the overlay between the top grating and the asymmetric part of the bottom grating; and $S_{asy}$ is the position of the asymmetric part of the bottom grating to the reference point. The former is the correct overlay of the top and bottom gratings without the affection of the asymmetric structure, and the latter is the overlay error caused by the asymmetric bottom grating profile structure. For clarity, Figure 3 shows the relations between $S_{rect}$, $S_{G1}$, $S_{asy}$, $OVL_{ori}$, and $OVL_{err}$. The ${\mathrm{LW}}_{\mathrm{BG}}$ represents the linewidth of the bottom grating in Figure 3.

### 2.3. Quasi-Period Linewidth Design

The second term in Equation (26) is an additional positive/negative first-order light intensity difference due to the asymmetric bottom grating structure. Hence, if we can make the second term in Equation (26) equal to zero, the measured overlay value under an asymmetric bottom grating structure would be as accurate as the overlay value measured under a perfect bottom grating structure.

There are three main factors in the second term of Equation (26), i.e., $\gamma_{2}$, $sin\left(\beta \right)$, and $sin\left(2\pi OVL_{err}/P\right)$. The first factor, $\gamma_{2}$, is a function of $t_{G1}$, $r_{G1}$, $r_{asy}$, and $t_{f}$. However, none of these items would be zero under an asymmetric bottom grating structure. Moreover, β is the phase term that is related to the middle-film structure and cannot meet the criteria that $sin\left(\beta \right)=0$ unless there is a unique design for the middle-film structure. Fortunately, the $OVL_{err}$ is a controllable factor, which can be adjusted by varying the linewidth of the bottom grating. Hence, it is possible to make the third factor zero, and it can be expressed as

(27) $$sin\left(\frac{2\pi }{P}OVL_{err}\right)=0.$$

From Equation (27), we have

(28) $$\frac{2\pi }{P}OVL_{err}=0,\;\pm \pi ,\;\pm 2\pi ,\cdots .$$

**Figure 4:**
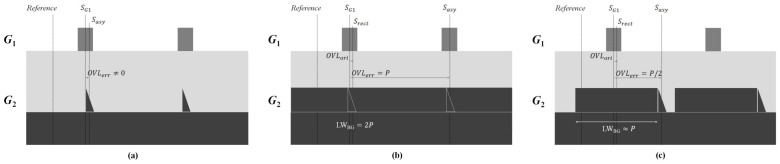
Graphical explanation of the overlay target design to satisfy $sin\left(2\pi OVL_{err}/P\right)=0$. (**a**) $2\pi OVL_{err}/P=0$ so that ${\mathrm{LW}}_{\mathrm{BG}}=0$ is not allowable; (**b**) $2\pi OVL_{err}/P=\pm 2\pi$ so that ${\mathrm{LW}}_{\mathrm{BG}}=2P$ is not allowable; (**c**) $2\pi OVL_{err}/P=\pm \pi$ so that ${\mathrm{LW}}_{\mathrm{BG}}\approx P$ is allowable.

Next, we discuss three different cases for Equation (28). The first case is when $\frac{2\pi }{P}OVL_{err}=0$. In addition, the associated criteria is $OVL_{err}=0$, which is not possible, since the ${\mathrm{LW}}_{\mathrm{BG}}\neq 0$, so that $S_{rect}\neq 0$, and then $S_{asy}\neq 0$ and $OVL_{err}=S_{asy}-S_{G1}\neq 0$, as shown in Figure 4a.

The second case is when $\frac{2\pi }{P}OVL_{err}=\pm 2\pi$, and the associated criteria is $OVL_{err}=\pm P$ and the ${\mathrm{LW}}_{\mathrm{BG}}=2P$. The linewidth of the bottom grating cannot be greater than the grating period or the grating lines will be merged, as shown in Figure 4b. Hence, the criteria $OVL_{err}=\pm P$ is not allowable.

The third case is when $\frac{2\pi }{P}OVL_{err}=\pm \pi$, and the associated criteria is that $OVL_{err}=\pm P/2$. As shown in Figure 4c, if $OVL_{err}=P/2$, the linewidth of the bottom grating will be nearly equal to or less than P, which is allowable in the semiconductor design rule. Under this criterion, the $OVL_{err}=S_{asy}-S_{G1}\approx \;P/2$, so that $sin\left(\frac{2\pi }{P}OVL_{err}\right)=0$.

Consequently, the only workable solution to minimize the overlay error caused by the asymmetric sidewall structure is a quasi-period linewidth overlay target design in which the linewidth of the bottom grating is nearly equal to or less than P.

## 3. Simulation Setup and the Simulated Asymmetric Structure

### 3.1. Simulation Conditions Setup

In this paper, the finite-difference time-domain (FDTD) methodology was used to simulate the $OVL_{err}$ for the DBO target with an asymmetric structure. In the simulation, only one unit cell of the overlay mark was used. Periodic boundary conditions (PBCs) were set on the right and left sides. Moreover, a perfectly matched layer (PML) was set under the simulation mark to absorb all the transmitted energy of the simulation mark. The wavelengths of the incident light in the simulation settings were from 400 nm to 700 nm with 5 nm interval so that 61 wavelengths were simulated, and the incident angle was 45°. For simplicity, the polarization was set to be S-polarization, i.e., parallel to the grating direction.

**Figure 5:**
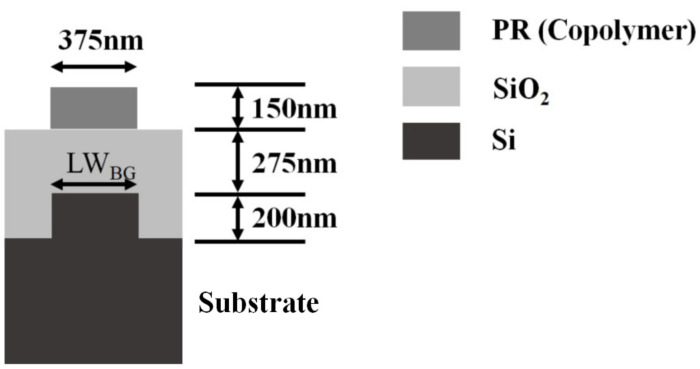
The film structure used for simulation.

Figure 5 shows the film structure used for simulation. The material of the substrate was Si, and the material of the bottom grating was Si with a height of 200 nm. A SiO_2_ film with 275 nm thickness was on the top of the bottom grating, and the material of the top grating was copolymer resist [28]; its period was the same as that of the bottom grating, and the height and the line width were 150 nm and half pitch, respectively.

In FDTD simulations, the setting of the mesh grid size affects the accuracy of the simulation results. Based on previous research [20], we set the mesh grid size to be 2 nm in the global area, and for the asymmetric structure area, the mesh grid size was set to be 0.1 nm to improve the simulation accuracy. The OVL range of this modified setting was also less than $1.5\times 10^{-4}\;\mathrm{nm}$, and the time required for one simulation could be reduced to about 360 s.

### 3.2. Sidewall Asymmetric Structure Setup for Simulation

**Figure 6:**
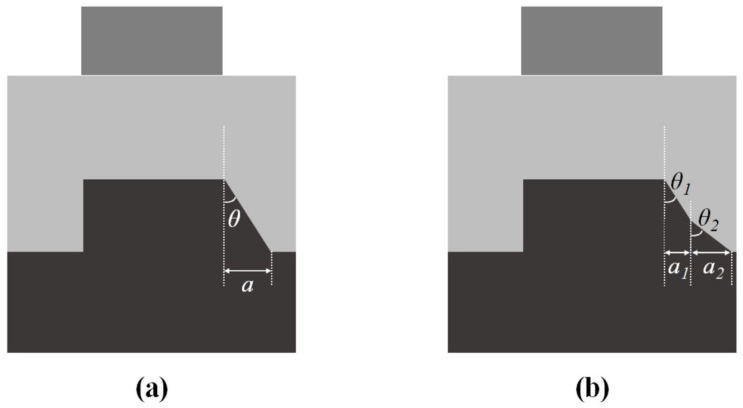
The schematic diagram of the asymmetric structure: (**a**) the simple sidewall asymmetric bottom grating structure; (**b**) the two-level sidewall asymmetric bottom grating structure.

The general and simplest sidewall asymmetric (SWA) bottom grating structure is shown in Figure 6a. The left and right grating sidewalls were tilted asymmetrically; *θ* was defined as the asymmetric angle of the left and right sides of the grating, and the asymmetric length a was the difference in length of the left and right sides protruding outward at the bottom of the two sides of the grating. In a perfect situation, which means no asymmetry, we would have θ=0 and a=0. However, the within-wafer uniformity of SWA should be considered in an actual situation. Hence, the SWA θ we used in the simulation was from 0° to 1.7°, and the corresponding *a* value was from 0 nm to 6 nm.

Next, a two-level sidewall asymmetric bottom grating structure was used to demonstrate the generalization usage of this methodology. Figure 6b shows the two-level SWA bottom grating structure, which was composed of two different asymmetric angles, namely, $\theta_{1}$ and $\theta_{2}$, and the associated asymmetric lengths $a_{1}$ and $a_{2}$. The intersection of the two different SWAs was set to be the middle height of the bottom grating. The corresponding values of $a_{1}$ and $a_{2}$ were from 0 nm to 6 nm.

## 4. Simulation Results and Discussions

### 4.1. Simple Sidewall Angle Asymmetric Structure

**Figure 7:**
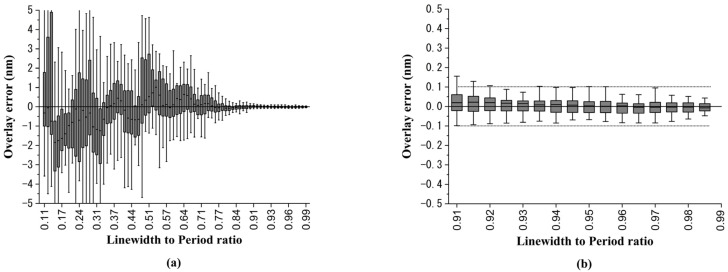
Overlay error simulation results for sidewall angle asymmetric structure: (**a**) the overlay error versus linewidth-to-pitch ratio box plot, and (**b**) the overlay error under linewidth-to-pitch ratios from 0.91 to 0.99.

In the first case, we considered a DBO target with a simple sidewall angle asymmetric structure for the bottom grating with the asymmetric length a value from 0 nm to 6 nm. The grating period in the simulation was set to be 750 nm. As shown in Figure 7a, the horizontal axis represents the different linewidth-to-period ratios of the bottom grating. The larger the value, the closer the grating line width is to the grating period. The vertical axis represents the magnitude of the OVL error obtained by the simulation. Since there were 61 wavelengths in the simulation setting (400 nm to 700 nm with 5 nm intervals), a box plot was used to represent the OVL error distribution for 61 wavelengths. It can be seen from Figure 7a that when the linewidth-to-period ratio was closer to 1, the OVL error was closer to 0. Figure 7b shows the partial magnification when the linewidth-to-period ratios were from 0.91 to 0.99, and when the linewidth-to-pitch ratio was greater than 0.93, the OVL error of each wavelength could be less than ±0.1 nm.

**Figure 8:**
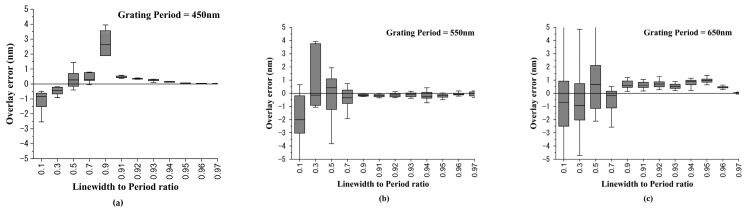
Overlay error simulation results under different grating periods. The grating periods were set to be (**a**) 450 nm; (**b**) 550 nm; and (**c**) 650 nm.

**Figure 9:**
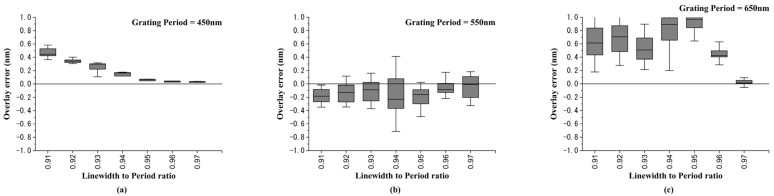
The overlay error under linewidth-to-pitch ratios from 0.91 to 0.97 under different grating periods. The grating periods were set to be (**a**) 450 nm; (**b**) 550 nm; and (**c**) 650 nm.

When the DBO target is being designed, the designer can change the grating period. Therefore, in the following case we considered changing the grating period from 750 nm to 450 nm with an interval of 100 nm. Figure 8a–c show the overlay error box plot with the linewidth-to-period ratio changing from 0.1 to 0.97 for grating periods of 450 nm, 550 nm, and 650 nm, respectively. The simulation results showed that the OVL error could be converged and reduced for different grating periods when the linewidth-to-period ratios were greater than 0.91. Figure 9a–c show the overlay error box plots with linewidth-to-period ratios changing from 0.91 to 0.99 for grating periods of 450 nm, 550 nm, and 650 nm, respectively. The simulation results of all simulated grating periods showed that the overlay error could be less than ±1 nm when the linewidth-to-period ratios were greater than 0.91. Moreover, when the linewidth-to-period ratios were equal to 0.97, the overlay error could be less than ±0.2 nm.

### 4.2. Film Thickness Variation Simulation

**Figure 10:**
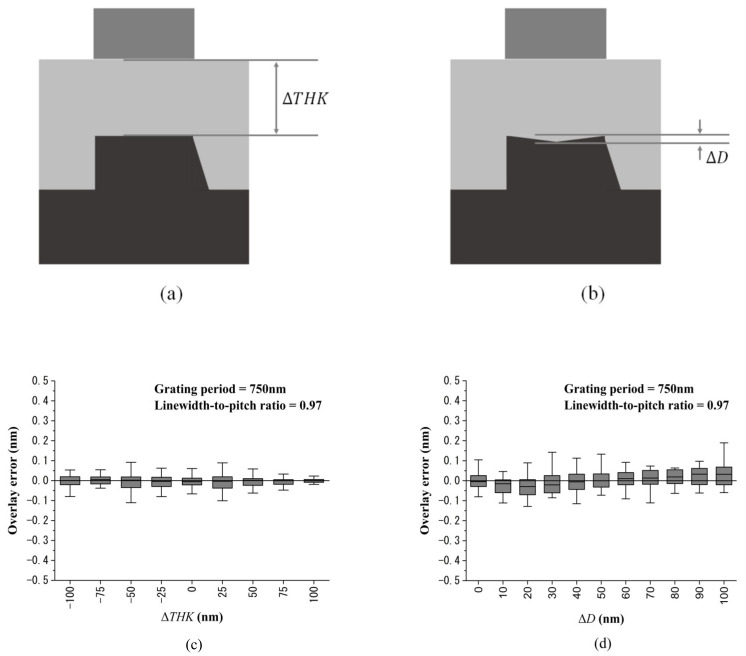
The film thickness variation simulations. The two cases of thickness variations: (**a**) simple thickness variation of SiO_2_; (**b**) the sunken top profile of the bottom grating. The corresponding box plot of the overlay error simulation results of (**c**) the first case and (**d**) the second case.

In the actual semiconductor process, the coating thickness varies within a wafer. Therefore, it is necessary to consider the influence of thickness variation. In this study, we considered two cases of thickness variations. The first case was simply the thickness variation of SiO_2_, as shown in Figure 10a. For convenience, it was assumed that the thickness variation of SiO_2_ was ΔTHK=±36% (i.e., 275 nm ± 100 nm), the grating period was set to be 750 nm, and the linewidth-to-pitch ratio of the bottom grating was set to be 0.97. The second case was the top profile of the bottom grating that was sunken by the CMP process, as shown in Figure 10b. It was assumed that the sunken profile was symmetric, and the sunken depth was ΔD=0~100 nm, and the settings of the grating period and the linewidth-to-pitch ratio were the same as those in the first case. Figure 10c shows the box plot of the overlay error under different thickness variations of the first case. Since the linewidth-to-pitch ratio of the bottom grating was set to be 0.97, the overlay error caused by the asymmetric sidewall structure was minimized. As seen in Figure 10c, even when the thickness of SiO_2_ varied by ±36%, the values of the overlay error could still be controlled within ±0.1 nm. For the overlay error simulation results of the second case, as shown in Figure 10d, the values of the overlay error could still be controlled within ±0.2 nm under the sunken depth of ΔD=0~100 nm.

### 4.3. Two-Level Sidewall Asymmetric Bottom Grating Structure

**Figure 11:**
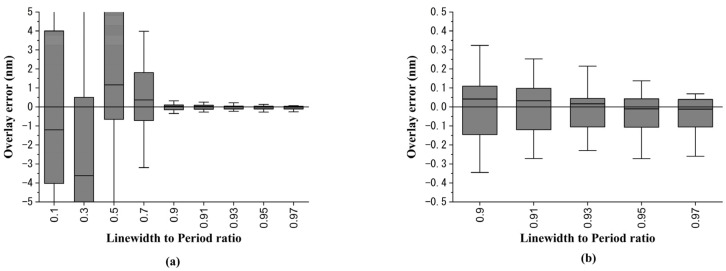
Overlay error simulation results for sidewall angle asymmetric structure: (**a**) the overlay error versus linewidth-to-pitch ratio box plot; (**b**) the overlay errors under linewidth-to-pitch ratios are from 0.91 to 0.99.

Next, a two-level asymmetric sidewall bottom grating structure, which is shown in Figure 6b, was used to demonstrate the generalization usage of the asymmetric sidewall structure of this methodology. For simplicity, the grating period was set to be 750 nm. The simulated overlay error versus the linewidth-to-pitch ratio of the bottom grating is shown in Figure 11. As seen in Figure 11a, as per our expectations, the overlay error decreased as the linewidth-to-pitch ratio of the bottom grating became larger. The overlay error was controlled within ±0.3 nm, as the linewidth-to-pitch ratio of the bottom grating was greater than 0.91, as shown in Figure 11b.

## 5. Conclusions

In this paper, we first derived an overlay calculation formula for the DBO measurement with a symmetric bottom grating structure of the overlay target based on basic interference theory. For the overlay target with an asymmetric sidewall profile of the bottom grating structure, we separated the asymmetric structure into two parts: the symmetric and the asymmetric parts. Then, the overlay error term caused by the asymmetric part was deduced. Based on the derived results, we proposed an improved overlay target design by increasing the linewidth of the bottom grating closed to the grating period to minimize the overlay error caused by the sidewall asymmetry structure. An FDTD simulation was used to demonstrate the feasibility of the overlay target design proposed by this method.

In the first case of the simulation, simple sidewall asymmetry was tested. The simulation results showed that under different grating periods, as the linewidth to period ratio of the bottom grating increased and became greater than 0.9, the overlay error decreased within ±0.3 nm. This overlay error was accurate enough for the 5 nm technology node. Moreover, the simulation showed that if the linewidth-to-period ratio of the bottom grating was set to be 0.97, the overlay error could be within ±0.1 nm. Then, the different film thickness variations simulation demonstrated the capability of the proposed overlay target design, and the overlay error could be controlled within ±0.1 nm, even when the thickness of SiO_2_ varied by ±36%. In the last case, a two-level sidewall asymmetric structure was tested. The simulation results showed that the overlay error was controlled within ±0.3 nm as the linewidth-to-pitch ratio of the bottom grating was greater than 0.91.

Moreover, the overlay error must be further improved for an incoming 1 nm or angstrom meter technology node. Since different pitches of the overlay target design result in different overlay errors, as shown in Figure 8 and Figure 9, one could further improve the overlay error by designing an overlay target with a suitable pitch.

## Data Availability

Not applicable.
